# Farnesoid X receptor activation promotes cell proliferation via PDK4-controlled metabolic reprogramming

**DOI:** 10.1038/srep18751

**Published:** 2016-01-05

**Authors:** Yang Xie, Hong Wang, Xuefang Cheng, Yuzheng Wu, Lijuan Cao, Mengqiu Wu, Wen Xie, Guangji Wang, Haiping Hao

**Affiliations:** 1 State Key Laboratory of Natural Medicines, Key Laboratory of Drug Metabolism and Pharmacokinetics, China Pharmaceutical University, Nanjing 210009, China; 2 Center for Pharmacogenetics and Department of Pharmaceutical Sciences, University of Pittsburgh School of Pharmacy, Pittsburgh, Pennsylvania 15261, USA

## Abstract

Farnesoid X receptor (FXR) plays a pivotal role in the regulation of various metabolic pathways as well as liver regeneration. However, the casual link between cell proliferative effects during liver regeneration and metabolic regulation of FXR was elusive. In this study, we found that FXR activation significantly promotes HepG2 cell proliferation accompanied with metabolic switch towards the excessive accumulation of aerobic glycolytic intermediates including lactic acid, pyruvate and the subsequently increased biosynthesis of glycine. This FXR-induced metabolic switch was found dependent on an up-regulation of pyruvate dehydrogenate kinase 4 (PDK4), a FXR target gene. FXR agonists were found to promote liver regeneration in the murine model of APAP induced liver injury, which was associated with a metabolic switch favoring the accumulation of glycolytic intermediates as precursors for generation of biomass. However, FXR activation has little effect on the glycolytic metabolism in healthy primary hepatocytes *in vitro* and the liver of healthy mice *in vivo*. Therefore, we conclude that FXR may promote the proliferation of tumor cells and the hepatocytes in the process of liver regeneration by activating the PDK4-mediated metabolic reprogramming to generate glycolytic intermediates essential for rapid biomass generation, establishing a mechanistic link between cell proliferation and metabolic switch.

The farnesoid X receptor (FXR, NR1H4) is a ligand-activated transcription factor that belongs to the nuclear receptor (NR) superfamily^1^. FXR regulates the expression of various genes involved in the metabolism of bile acid, lipid and glucose^2^,^3^,^4^. Recent studies showed that FXR also plays a role in liver regeneration, a compensatory regrowth of liver following liver damage. Specifically, liver regeneration after partial hepatectomy was impaired in Fxr^−/−^ mouse^5^,^6^. Besides, Fxr^−/−^ mouse was found more sensitive to carbon tetrachloride (CCl_4_)^7^ and acetaminophen (APAP) induced hepatotoxicity^8^. More recently, it was found that FXR activation directly promoted proliferation of various cancer cell lines. These results suggest that FXR is an important player in regulating cell proliferation in response to various stimuli. Although several mechanisms, such as the suppression of p16/INK4a^9^,^10^, have been proposed to account for FXR induced liver regeneration, a direct causal link between FXR activation and cell proliferation remains established.

Similar to the widely accepted Warburg effect in tumor cells, proliferating mammalian cells also exhibit an adaptive increase in aerobic glycolysis to facilitate the uptake and incorporation of nutrients into the biomass needed to produce new cells^11^,^12^. The increased production of glycolytic intermediates is shunted into subsidiary pathways to fuel metabolic pathways that generate de-novo nucleotides, lipids, amino acids, and NADPH^13^,^14^. As a nuclear receptor, FXR plays an intriguing role in regulating glucose metabolism. FXR inhibits gluconeogenesis regulating the expression and activity of PPAR γ coactivator 1α (PGC1α)^15^ and phosphoenolpyruvate carboxykinase (Pepck)^16^, and also regulates the expression and function of PDKs, which are key enzymes in glycolysis pathway^17^. We thus hypothesized that activation of FXR may promote cell proliferation and liver regeneration by controlling a metabolic switch favoring the generation of biomass.

To test our hypothesis, a GC/MS based metabolomics analysis together with isotope tracer was utilized to investigate the metabolic change associated with the FXR-induced cell proliferation and liver regeneration using the APAP injury model mice. We show that FXR activation by both chenodeoxycholic acid (CDCA) and GW4064 significantly promotes proliferation of HepG2 cells, which largely depends on PDK4 regulated metabolic adaptation to generate excessive glycolytic metabolites and to promote glycine biosynthesis. *In vivo*, a compensatory activation of FXR was observed in mice in response to APAP-induced liver injury, which was accompanied by a similar metabolic switch as that observed *in vitro*.

## Results

### FXR promotes proliferation of tumor cell lines

**Figure 1:**
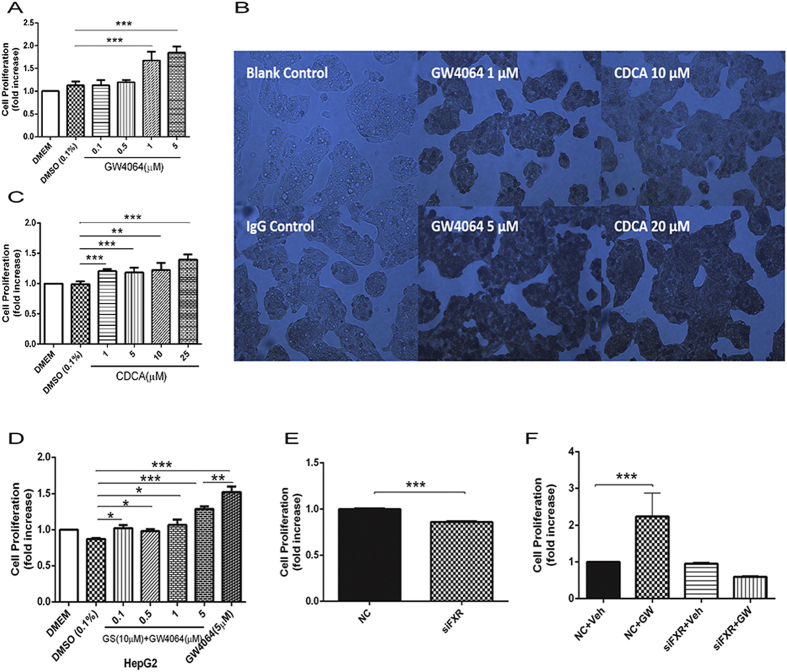
FXR promotes proliferation of tumor cell lines. (**A–C**) Dose-dependent effect of FXR agonists in the proliferation of HepG2 cells. (**A**) HepG2 cells were treated with different concentrations of GW4064. (**B**) The proliferation of HepG2 cells was investigated using the BrdU Proliferation Kit after treatment with GW4064 or CDCA. (**C**) HepG2 cells were treated with different concentration of CDCA. (**D**) Dose-response for FXR agonist GW4064 combined with the treatment of 10 μΜ FXR antagonist (Z)-Guggulsterone (GS). HepG2 cells were treated with 10 μΜ GS together with different concentrations of GW4064. (**E**) HepG2 cells were treated with FXR siRNA or negative siRNA. (**F**) HepG2 cells were treated with FXR siRNA or negative siRNA before treatment with GW4064 or vehicle. Values of all MTT assays are presented as mean ± SD (n = 6; *P < 0.05; **P < 0.01; ***P < 0.001).

Since the mechanism by which FXR promotes liver regeneration and cell proliferation is not fully understood, we sought to determine whether FXR may influence cell proliferation directly. In this experiment, the effects of FXR activation on the cell proliferation of various tumor cell lines including HepG2, SK-Hep1, and HT29, as well as primary hepatocytes, were tested by using selective FXR agonists GW4064 or CDCA^18^,^19^. Treatment of both agonists significantly promoted the proliferation of all three tumor cell lines (Fig. 1A,C, Fig. S1) in a dose-dependent manner. Moreover, the BrdU assay of DNA synthesis supported that GW4064 or CDCA treatment markedly promoted the proliferation of HepG2 cells (Fig.1B). FXR antagonist (Z)-Guggulsterone (GS) pretreatment was shown to effectively inhibit the cell proliferation induced by GW4064 (Fig. 1D). FXR short interference RNA (siRNA) treatment significantly suppressed both the basal (Fig.1E) and GW4064- inducible (Fig. 1F) proliferation of HepG2 cells. All these results support that the activation of FXR promotes the proliferation of cancer cell lines. In contrast, FXR activation had little effect on the proliferation of primary mice hepatocytes (Fig. S2A)

### Activation of FXR triggers metabolic reprogramming

**Figure 2:**
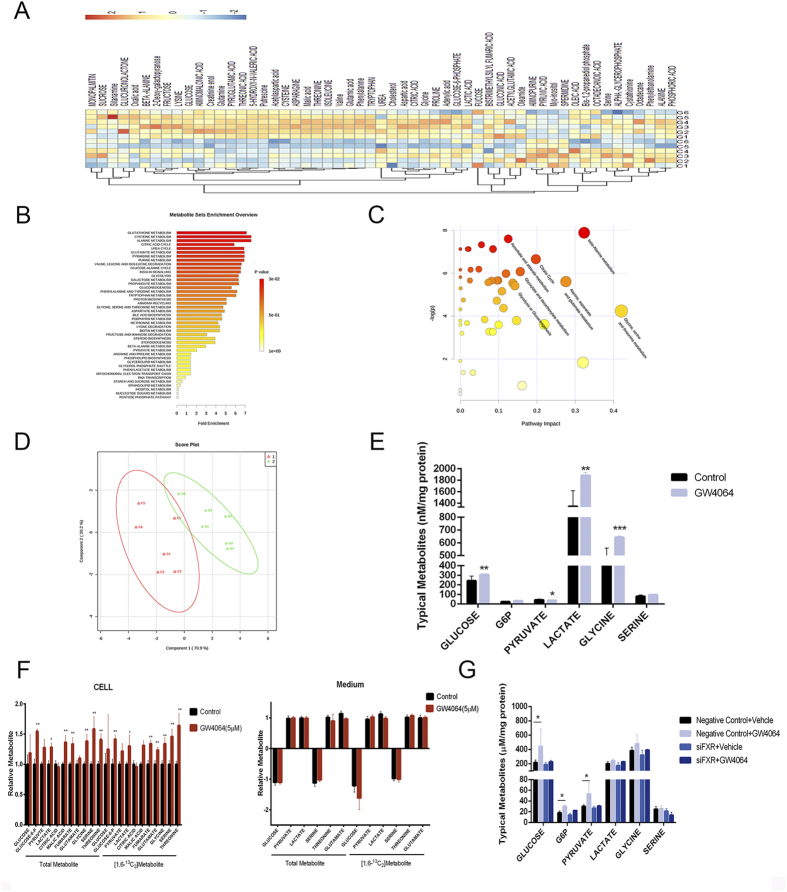
Activation of FXR triggers metabolic reprogramming. (**A**) Heatmaps of HepG2 cells treated with GW4064. Control Group, C1 to C6; GW4064-treated Group, G1 to G6. (**B,C**) The impact of GW4064 on HepG2 cell metabolic pathways. Intracellular metabolite-based metabolic pathway analysis of HepG2 cells (**B**) Overview of metabolites that were enriched in HepG2 cells based on HepG2 cell intracellular metabolites (**C**). (**D**) PLS-DA scoring plot based on GC/MS metabolic profiles (R2X = 0.722; R2Y = 0.852; Q2Y = 0.738). (**E**) Concentrations of major intracellular intermediates in glycolysis pathway in HepG2 cells. (**F**) Relative metabolite abundance in HepG2 cells grown in [1, 6-^13^C_2_]-glucose upon treatment of GW4064. Data are presented as the total metabolite pool and the ^13^C-labelled and Glc-derived metabolite pool (right). (**G**) Concentrations of major intracellular intermediates in glycolysis pathway in HepG2 cells when treated with FXR siRNA with or without the combined treatment of GW4064. Values are presented with mean ± SD (n = 6; *P < 0.05; **P < 0.01; ***P < 0.001).

FXR activation by GW4064 and CDCA induced a significant metabolic shift from the control cells, while the siRNA knock-down of FXR could largely abolish such a shift (Fig.2A–D). Detailed metabolites analysis suggested disturbed levels of dozens of metabolites induced by GW4064 and CDCA treatment (Table S1). To assess the metabolic pathways of marker metabolites associated with FXR activation, enrichment analysis and pathway analysis were performed using MetaboAnalyst 2.0. The results showed that the glucose metabolism and amino acid metabolism pathways were significantly disturbed by GW4064 (Fig. 2B). The detailed analysis showed that the activation of FXR by GW4064 resulted in a significant increase in production of most glycolysis metabolites, such as lactic acid, pyruvate acid, and glucose-6-phostate (Fig. 2E). Isotope tracer experiment using ^13^C_2_-glucose further proved that FXR activation resulted in the enhancement of glucose uptake as well as glycolysis (Fig. 2F). CDCA treatment induced quite similar trends in the metabolic reprogramming of HepG2 cells, supporting a common metabolic shift induced by activation of FXR (Table S1). Accordingly, FXR knock-down by siRNA transfection led to a significant inhibition and even the further decrease in production of lactic acid, pyruvate acid, glucose-6-phostate, the uptake of glucose, as well as the production of glycine and serine triggered by GW4064 treatment (Fig. 2G). In line with the cell proliferation assay, FXR activation had negligible effects on the metabolic profile in primary hepatocyte (Fig. S2B).

### PDK4 mediates the FXR responsive metabolic reprogramming

**Figure 3:**
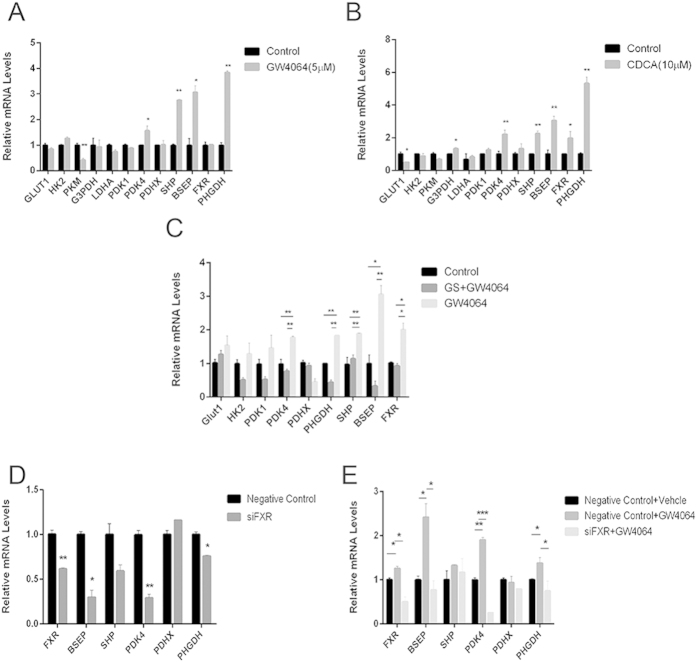
PDK4 mediates metabolic reprogramming induced by FXR activation. (**A**) Expression of major enzymes in the glycolysis pathway in HepG2 cells after treatment with GW4064. (**B**) Expression of major enzymes in the glycolysis pathway in HepG2 cells after treatment with CDCA. (**C**) Expression of major enzymes in the glycolysis pathway in HepG2 cells after treatment with 10 μM GS combined with 5 μM GW4064. (**D**) Expression of major enzymes in the glycolysis pathway in HepG2 cells after transfection with FXR siRNA. (**E**) Expression of major enzymes in the glycolysis pathway in HepG2 cells transfected with FXR siRNA with or without GW4064 treatment. Values are presented with mean ± SD (n = 6; *P < 0.05; **P < 0.01; ***P < 0.001).

We then performed a series analysis of various enzymes involved in the related pathways. Because GW4064 or CDCA induced similar metabolic shifts and cell proliferative effects, we sought to identify the common enzymes which are disturbed by both agents. As shown in Fig. 3A,B, PDK4, involved in phosphorylation and inactivation of pyruvate dehydrogenase complex, was observed with a significant up-regulation in HepG2 cells treated with either GW4064 or CDCA. PHGDH, responsible for the first step of the serine biosynthetic pathway downstream of glycolysis, was also observed with a 4-fold up-regulation after FXR agonist treatment. Meanwhile, treatment with GS or FXR siRNA reduced the expression of PDK4 and PHGDH, and abrogated GW4064 induced up-regulation in HepG2 cells (Fig. 3C–E). The gene expression profile coincides with the metabolic alterations, hinting to a critical role of PDK4 and PHGDH in mediating the metabolic reprogramming and proliferative effects of FXR activation in cancer cell lines but not the primary hepatocytes (Fig. S2C).

**Figure 4:**
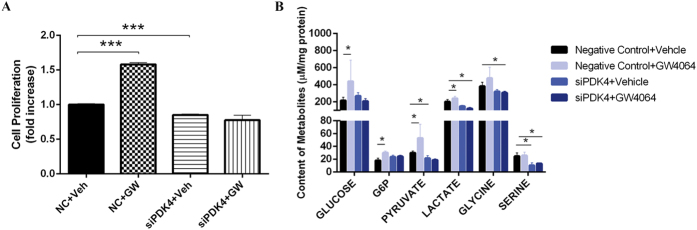
PDK4 mediates FXR activation-induced cell proliferation and promotion of glycolysis. (**A**) Cell proliferation of HepG2 cells when transfected with PDK4 siRNA or control siRNA (NC). (**B**) Relative abundance of major intracellular intermediates in glycolysis pathway in HepG2 cells when treated with PDK4 siRNA with or without the combined treatment of GW4064. Values are presented with mean ± SD (n = 6; *P < 0.05; ***P < 0.001).

Because the up-regulation of PDK4 may inactivate pyruvate dehydrogenase complex and shift the metabolic use of pyruvate from energy production via TCA cycle to biomass generation, we hypothesized that PDK4 might be an important mediator in the cell proliferative effect of FXR activation. As expected, PDK4 knock-down by siRNA markedly abrogated the cell proliferative effect of GW4064 (Fig. 4A). PDK4 siRNA transfection also abolished the metabolic reprogramming effect upon FXR activation. The cellular accumulation of glycolytic intermediates including pyruvate, lactate, and G6P induced by GW4064 was largely abolished by PDK4 knockdown (Fig. 4B). These results suggested that PDK4 mediated the FXR-responsive cell proliferation and metabolic reprogramming.

### FXR promotes liver regeneration after APAP injury

**Figure 5:**
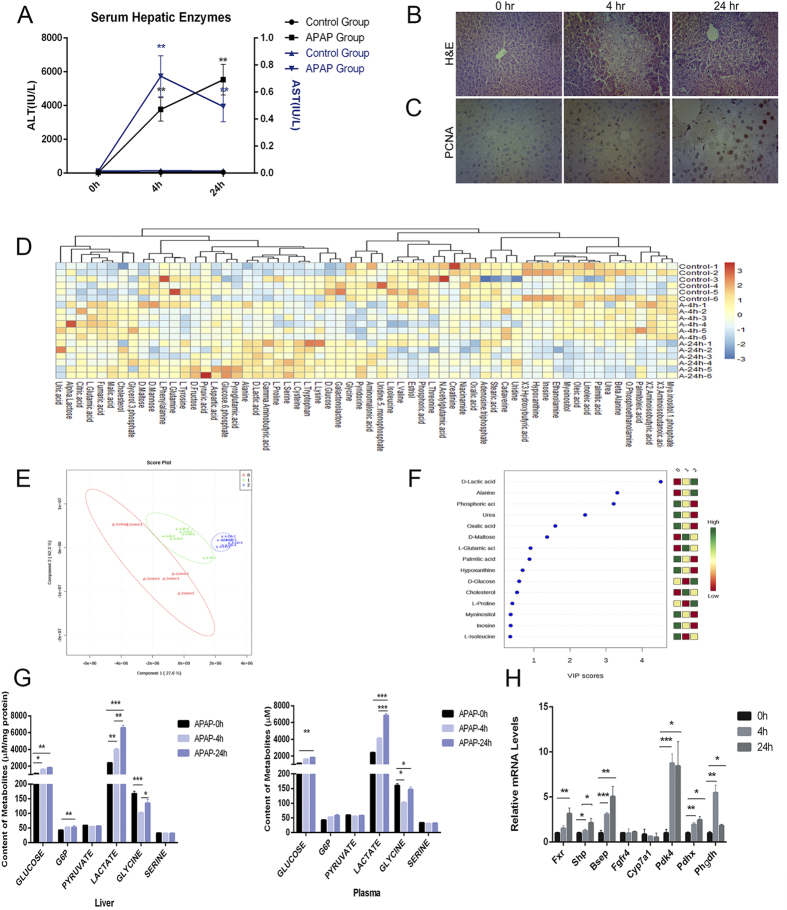
FXR promotes liver regeneration after APAP injury. (**A**) Serum ALT and AST. (**B**) Representative photomicrographs of H&E-stained liver sections. Original magnification, × 200. (**C**) Representative photomicrographs of PCNA IHC staining of liver sections of APAP-treated mice. Original magnification, ×200. (**D**) Heatmaps of liver samples treated with GW4064. (**E**) PLS-DA scoring plot of GC/MS metabolic profiles (R2X = 0.667; R2Y = 0.944; Q2Y = 0.921). (**F**) Variable importance in projection (VIP) in PLS-DA model. (**G**) Concentrations of major intermediates in glycolysis pathway in liver and plasma. (**H**) Gene expression of FXR target genes, *Pdk4, Pdhx* and *Phgdh* in liver after APAP treatment of mice. Values are presented with mean ± SD (n = 6; *P < 0.05; **P < 0.01; ***P < 0.001).

Our results showed that FXR activation promoted the proliferation and metabolic adaptation of cancer cell lines but not the primary hepatocytes. It is well known that hepatocytes are able to proliferate in the process of liver regeneration when the liver is damaged by various factors. We thus asked whether FXR activation could also promote the proliferation of hepatocytes in the process of liver regeneration. It had been previously reported that FXR activation could protect against APAP induced liver injury but the mechanisms remain largely elusive. We thus employed an APAP injury mouse model to examine the role of FXR activation in the promotion of liver regeneration and its association with metabolic reprogramming. The time-course study of APAP toxicity by histological and biochemical analysis suggested that APAP induced a very rapid liver injury with significant recovery at 24 hours (Fig.5A,B). PCNA immunostaining revealed that the liver cell proliferation was more vigorous at 24 h than that at 0 or 4 h after APAP intoxication (Fig. 5C). GC/MS based metabolomics analysis indicated a time-dependent shift of hepatic and plasma metabolites after the APAP injury (Fig. 5D,E). The Variable Importance in Projection analysis (Fig. 5F) of liver biomarker metabolites showed that multiple metabolites in the pathways of glucose metabolism, especially lactic acid, were significantly enhanced. Analysis of the concentrations of major metabolites in glycolysis pathway in liver and plasma confirmed the accumulation of glycolytic intermediates during liver repair (Fig. 5G). It was interesting to note that the accumulation of lactic acid was more significant at 24 h than that at 4 h after APAP injury, suggesting that the accumulation of lactic acid is a possible biomarker of liver regeneration. Accompanied by the metabolic changes, the expression of FXR and its downstream target genes was found up-regulated 24h after APAP-treatment (Fig. 5H), suggesting an adaptive response of injured liver to activate FXR in favor of spontaneous liver repair. Consistent with the metabolic profiles, a significant up-regulation of *Pdk4* and *Phgdh* was observed as early as 4 h after APAP treatment (Fig. 5H).

**Figure 6:**
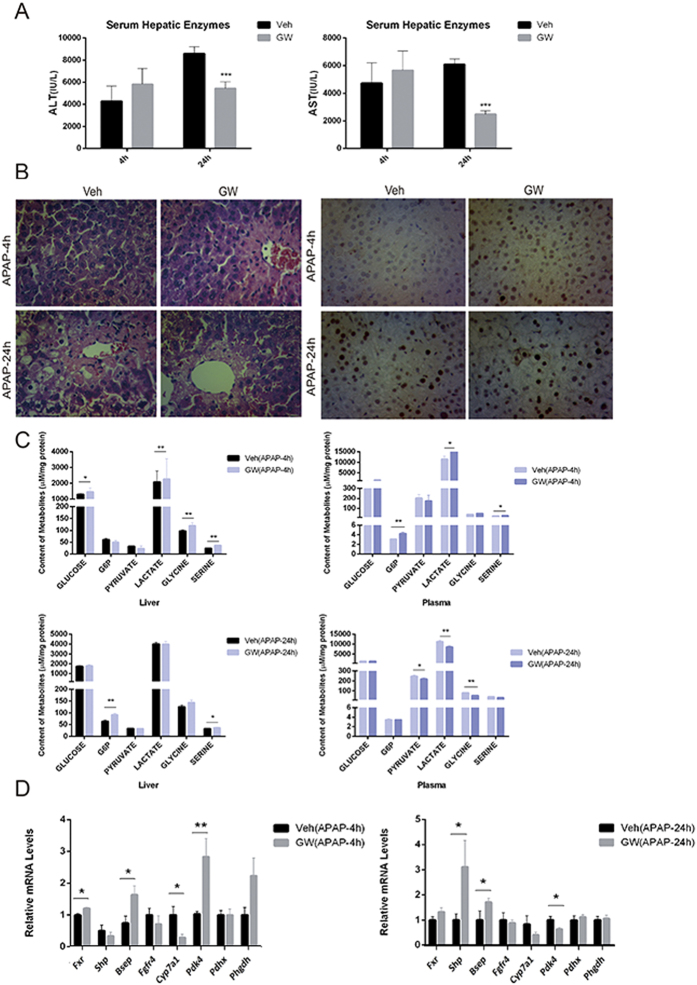
Activation of FXR advances liver regeneration associated with glycolytic reprogramming. Mice were pretreated with vehicle (Veh) or GW4064 (GW) for 3 days and subsequently challenged with 300 mg/kg APAP. (**A**) Serum ALT and AST activity measured at 4 hours and 24 hours after APAP treatment of mice. (**B**) Representative photomicrographs of H&E-stained liver sections and photomicrographs of PCNA IHC staining of liver sections of APAP-treated mice. Original magnification, ×200. (**C**) Concentrations of major intermediates in glycolysis pathway in liver and plasma obtained at 4 hours and 24 hours after APAP treatment. (**D**) Gene expression of FXR target genes, *Pdk4, Pdhx* and *Phgdh* in liver. Values are presented with mean ± SD (n = 6; *P < 0.05; **P < 0.01).

To further validate the role of FXR activation in liver regeneration, mice were pretreated with vehicle or GW4064 (30 mg/kg) at 30 min before a single dose of APAP (300 mg/kg). Biochemical analysis of ALT and AST and the histological analysis supported a protective effect of GW4064 pretreatment against APAP induced liver injury (Fig. 6A,B). GW4064 pretreatment greatly promoted cell proliferation at 4 h after APAP treatment as evidenced by the increased expression of PCNA expression (Fig. 6C). As the metabolic level, GW4064 treatment led to a significant elevation of hepatic and plasma levels of glycolytic intermediates at 4 h but not at 24 h, which means an advanced metabolic reprogramming in favor of liver regeneration. Moreover, GW4064 treatment up-regulated the mRNA levels of *Pdk4* and *Phgdh* at 4 h but not at 24 h after APAP challenge (Fig. 6D). These results demonstrate that FXR activation may promote liver regeneration at an early stage and thereby protecting against APAP induced liver injury. In line with that observed from the primary hepatocytes study *in vitro*, FXR activation had little effects on the glycolytic metabolism in the liver of healthy mice (Fig. S3).

## Discussion

In this study, we showed that FXR activation significantly promoted proliferation of cancer cell lines and liver regeneration upon APAP injury. Mechanistically, we found that the cell proliferative effect induced by FXR activation both *in vitro* and *in vivo* is closely associated with PKD4 regulated metabolic switch towards the accumulation of glycolytic intermediates and the biosynthesis of glycine. Our results suggest a mechanistic link between the cell proliferative and metabolic adaptation effects regulated by FXR.

FXR is a member of the nuclear receptor superfamily playing a pivotal role in lipid and glucose metabolism, as well as liver regeneration and cell proliferation^5^,^6^,^20^. To test whether or not the cell proliferative effect is associated with the metabolic regulatory function upon FXR activation, we performed GC/MS based metabolomics analysis of the FXR responsive metabolic changes. We confirmed that FXR activation by both CDCA and GW4064 significantly promoted the proliferation of HepG2 cells, HT29 and SK-Hep1 cells. Pharmacological inhibition or genetic silencing of FXR abrogated the cell proliferative effect of FXR agonists, supporting an on-target effect of both agonists. Moreover, a typical FXR antagonist guggulsterone was found capable of inhibiting proliferation of a panel of cancer cells and is promising in the therapy of various solid tumors^21^,^22^. Therefore, this study clearly suggests a ligand dependent effect of FXR activation in the promotion of proliferation of cancer cells.

It has been widely accepted that rapid proliferating cells, such as cancer cells, involve a metabolic shift towards efficiently converting glucose and specific amino acids into biomass^12^,^23^. Previous studies suggest a potential relationship between FXR and glucose metabolism enzymes. Activation of FXR results in a down-regulation of glucongeogenesis acting through PGC1α^15^ and Pepck^16^,^24^. FXR was also shown to positively regulate the expression of PDK4^17^. However, no mechanistic link between the FXR-responsive cell proliferation and metabolic switch has been proposed. In this study, results from GC/MS based metabolomic analysis support that FXR activation drives an apparent metabolic shift towards an increased uptake of glucose and the accumulation of glycolytic intermediates such as G6P, lactate, and pyruvate, indicating a facilitated aerobic glycolysis even in the presence of oxygen. In addition, an increased synthesis of glycine was observed upon FXR activation, indicating a metabolic switch favoring the generation of biomass. Since CDCA and GW4064 possess similar characteristics in the promotion of cell proliferation and metabolic switch, we utilized this information to screen the same enzymes involved in the glycolytic pathways that are regulated by both CDCA and GW4064. As a result, significant up-regulation of PDK4 and PHGDH was observed upon FXR activation. PDK4, an isoform of PDKs, regulates the activity of PDHX, the gate keeping enzyme linking glycolysis to the TCA cycle and thereby preventing pyruvate use in the TCA cycle^25^,^26^. The observed enzyme regulation pattern coincides very well with the metabolic shift induced by FXR activation. We thus tested whether PDK4 controlled metabolic switch is involved in the cell proliferative effect by FXR activation. Indeed, a siRNA knockdown of PDK4 in HepG2 cells largely abrogated the FXR responsive cell proliferation and metabolic shift, indicating that PDK4 is an important downstream component in FXR activation triggered cell proliferation. Although the current study suggests that FXR activation and the downstream up-regulation of PDK4 can directly promote proliferation of cancer cells, it is important to note that the exact role of FXR and PDK4 in the tumor biology can be complex, and particularly, in the *in vivo* settings. PDK4 activation, as a downstream mechanism of PPARγ activation, may inhibit tumorigenesis *via* increasing ROS production^27^. Conversely, PDK4 activation may also promote tumorigenesis through activation of CREB-RHEB-mTORC1 signaling cascade^28^. FXR activation protects against tumorigenesis of various cancers while *Fxr-null* mice could spontaneously develop hepatocarcinogenesis, which can be largely explained by the function of FXR in maintaining homeostasis of bile acids^29^,^30^,^31^. Therefore, it seems that FXR activation may on one hand promote proliferation of cancer cells via facilitating a metabolic switch in favor of biomass generation, and on the other hand, may prevent carcinogenesis through restoring homeostasis of bile acids that are toxic and probably serving as carcinogens.

Since a pioneering finding of FXR-bile acids axis in driving homeotrophic liver growth^5^, the role of FXR activation in promoting liver regeneration has been extensively studied. As for the mechanisms of FXR activation in promoting liver regeneration, previous studies suggested a cell-autonomous effect of hepatic FXR in the up-regulation of cell proliferative genes such as Foxm1b and cyclin D1, and a bile acids-dependent effect that is largely attributed to intestinal FXR^6^,^20^,^32^. In this study, we found that the hepatotoxicity of APAP led to an adaptive activation of hepatic FXR and compensatory liver regeneration. Of particular interest, APAP induced and FXR activation driven liver regeneration were associated with a metabolic adaptation characterized with significantly elevated levels of glycolytic intermediates including lactic acid, pyruvate acid and glucose-6-phosphate in liver and plasma samples of mice, which is quite similar to that observed from the *in vitro* study of HepG2 cells. Together, our study indicates that the rapid proliferation of hepatocytes in the process of liver regeneration also involves a metabolic switch favoring the generation of biomass. Our results suggested that the FXR responsive cell proliferation and the metabolic switch is mechanistically linked in the process of liver regeneration.

**Figure 7:**
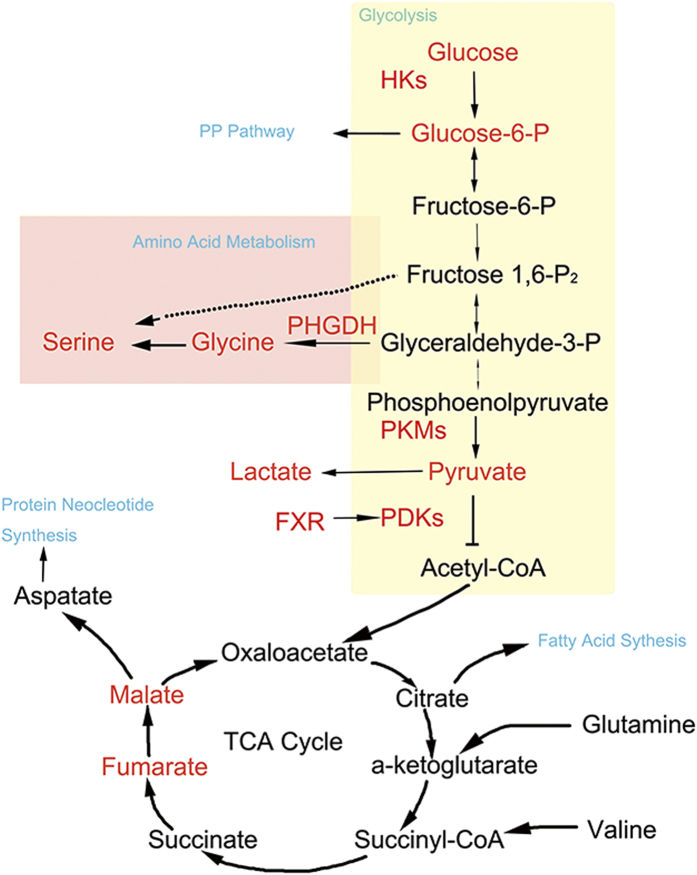
A scheme showing FXR activation triggered metabolic reprogramming in favor of cell proliferation. Activation of FXR results in increased expression of PDK4, which may lead to phosphorylation of PDHX and inhibit the pyruvate use in TCA cycle, leading to an increase in biosynthesis of glycine and serine, as well as other substrates needed to produce new cells.

In summary, we have shown that the activation of FXR results in increased expression of PDK4, which leads to phosphorylation of PDHX and inhibition of the pyruvate use in the TCA cycle, while switching to the subsequent elevation of PHGDH catalyzed serine biosynthesis, resulting in a metabolic reprogramming in favor of cell proliferation (Fig. 7). Our study establishes a mechanistic link between the metabolic reprogramming and cell proliferative effects of FXR activation and thereby providing a new insight to the understanding of the role of FXR activation in the process of liver regeneration.

## Methods

### Chemicals and reagents

Myristic-1,2-^13^C_2_ acid, 99 atom% ^13^C, the stable-isotope-labeled internal standard compound (IS), methoxyamine hydrochloride (purity 98%), and pyridine ( ≥ 99.8% GC) were purchased from Sigma-Aldrich (USA). Distilled water was produced by a Milli-Q Reagent Water System (Millipore, USA). D-Glucose-1,6-^13^C_2_ ( ≥ 99% isotope enrichment for each carbon position), GW4064, CDCA, (Z)-Guggulsterone (GS), and Acetaminophen were all from Sigma-Aldrich. BrdU cell proliferation kit were purchased from Keygene Biotech (Nanjing, China).

### Animals and Treatments

Specific pathogen free (SPF) male C57BL/6 mice (8 wk old, 20 g) were obtained from Comparative Medicine Centre of Yangzhou University, China. Animals were housed in an air-conditioned room (25 °C) under a 12 h light/dark cycle for 1 week before experiments and allowed water and standard chow *ad libitum*. The animal studies were approved by the Animal Ethics Committee of China Pharmaceutical University and have been carried out in accordance with the Declaration of Helsinki. Liver injury was induced by APAP as published before with little modification^8^,^33^. In the first batch of the experiment, mice were treated with a single intraperitoneal (*i.p*) injection of APAP (300 mg/kg) dissolved in warm sterile saline solution. In the second batch, mice were pretreated with vehicle or GW4064 (30 mg/kg, *i.p*) for 3 days once a day and a single *i.p* injection of APAP (300 mg/kg) was administered on the third day 0.5 h after the final dose of vehicle or GW4064. In all these experiments, mice were fasted for 14 h before APAP treatment and re-fed with normal food after APAP treatment. Tissues and serum were collected after 4 h and 24 h of APAP treatment. All tissues for GC/MS and mRNA analysis were snap frozen in liquid nitrogen and stored in −80 °C until further processed.

### Assessment of Liver Damage and Biochemical Analysis

For histological assay, tissue sections (5 μm) were stained with H&E. Liver sections were evaluated for necrosis scoring, as previously reported^34^. The pathological alterations were evaluated and confirmed by an experienced pathologist blinded to the experimental conditions. For the immunohistochemistry analysis, tissue specimens were fixed in 10% formalin for 12 to 24 h, dehydrated, and paraffin embedded. Standard immunohistochemical procedures were performed in detection of proliferating cell nuclear antigen (PCNA). For negative controls, 1% nonimmune pre-immune serum in PBS replaced the primary antibodies.

### Cell culture and treatment

Human liver carcinoma cell line, HepG2, was obtained from American Type Culture Collection (ATCC, USA). HepG2 cells were grown in Dulbecco modified Eagle’s medium (Gibico, USA) supplemented with 1 × MEM nonessential amino acids (Invitrogen, USA). Media were supplemented with 10 U/mL penicillin, 10 μg/mL streptomycin, and 10% fetal calf serum. Cells between passage 1 and passage 20 were incubated at 37 °C and 5% CO_2_.

CDCA (50 μM) and GW4064 (5 μM) were used as natural and synthetic FXR agonists. GS (10 μM) was used as natural FXR antagonists. For isotopic tracer assay, HepG2 cells were incubated in 1:1 [1,6 − ^13^C_2_]-glucose and unlabeled glucose at a final total glucose concentration of 25 mM in glucose-free Dulbecco’s Modified Eagle’s Medium (DMEM).

HepG2 cells were transfected with control siRNA or siRNA targeting FXR or PDK4 respectively using Lipofectamine 2000 (Invitrogen, USA) according to the manufacturer’s instruction. Cells were cultured for 48 h before treatment with 5 μM of GW4064 or vehicle for another 24 h. qRT-PCR analysis was conducted to examine the expression of genes in glycolysis pathway.

### Cell proliferation assay

Cells were grown in 96-well plates (1 × 10^4^/200 μL/well). After the first 24 h of culture, cells were treated for the next 24 h with GW4064 at the final concentrations of 0.1, 0.5, 1 and 5 μM, or CDCA at the final concentrations of 1, 5, 10, and 25μM. To further identify the role of FXR on cell proliferation, cells were treated with different concentrations of GW4064 in the presence of FXR antagonist GS (10 μM). After incubation for 24h, all cells were treated with 20 μL of MTT (5 mg/mL in PBS) per well for 3 h at 37 °C and then 150 μL of DMSO was added to dissolve the precipitated formazan and its absorbance was read in ELISA reader at 490 nm.

DNA synthesis was assessed by BrdU incorporation into HepG2 cells. Briefly, after treatment for 1 h with 30 μM BrdU, HepG2 cells were washed with PBS. The cells were then fixed with 4% formaldehyde for 30 min at room temperature, followed by removing the fixative and washing the cells in each well twice with PBS. The cells were then incubated with 2 M HCl for 15 min at room temperature followed by washed with 0.5% Tween-20/1% BSA/PBS. After incubated with 0.1 M NaB_4_O_3_ for 15 min at room temperature and washed with 0.5%Tween-20/1% BSA/PBS, cells were treated with 0.1% Triton X-100 for 2 min in ice cold plate for permeabilization. After treated with H_2_O_2_ solution for 5 min and Goat Serum Block Buffer for 1 h at 37 °C, the cells were then incubated with BrdU antibody overnight at 4 °C. Subsequently, the cells were treated with biotin-IgG for 60 min and streptomycin-HRP for 30 min at 37 °C. After washed with PBS, the cells were incubated with DAB for imaging.

### Organic solvent extraction of metabolites

For cell samples, the medium in each culture dish was discarded, and the adherent cells were quickly rinsed twice with cold isotonic saline (0.9% NaCl [w/v], 0.5 °C). Water (300 μL) was added to each dish, and then the dishes were stored in a freezer (−70 °C) before extraction. Cells were subjected to three freeze – thaw cycles of lysis (freezing for 60 min at −70 °C; thawing at 37 °C for 30 min), and after that, methanol containing 2.5 μg/mL of IS was added to the cell lysate in each dish. The adherent cells were then flushed with the solution ten times to ensure the thorough detachment of the cells. Finally, the suspension of cell debris in each dish was transferred to an Eppendorf tube, vigorously vortexed for 5 min, and then centrifuged at 20,000 × g for 10 min at 4 °C. To ensure that the same amounts of cell extracts were analyzed, the supernatants were quantitatively transferred to another tube, and evaporated to dryness in an SPD2010-230 Speed Vac Concentrator (Thermo Savant, USA).

For the extraction of medium and serum samples, 200 μL of methanol containing IS (2.5 μg/mL) was added to the samples (50 μL). Then the mixed solution was centrifuged at 20,000 × g for 10 min at 4 °C. For tissue samples, 800 μL of methanol containing IS (5 μg/mL) was added to liver samples (20 mg), then the mixture was homogenized and was centrifuged at 20,000 × g for 10 min at 4 °C. An aliquot of 100 μL supernatant was transferred to a GC vial following evaporation to dryness.

### Derivatization and GC/MS analysis

The methoximation, trimethylsylilation, and GC/MS analysis of the samples were as described previously^35^. Methylmyristate was added to the samples as the external standard (ES). Consequently, 0.5 μL of the derivatized sample was injected into the Shimadzu GC/MS system . The masses were acquired with m/z 50 – 700 at a rate of 30 spectra/s after a solvent delay of 170 s. Automaticpeak detection and mass spectrum deconvolution were performed with the Labsolutions (GCMS Solution Version 2.61), as reported previously^22^.

### Preparation of Total RNA and Quantitative Reverse Transcriptase PCR

Total tissue and cell RNA extraction was performed using the RNAiso Plus reagent (TaKaRa Biotechnology Co., Ltd, China) according to the manufacturer’s protocol. cDNA was generated from 500 ng total RNA using PrimeScript™ Reverse Transcriptase (TaKaRa Biotechnology Co., Ltd, China). qRT-PCR analysis was carried out using SYBR green PCR mastermix and analysed on a Bio-Rad CFX96 real time PCR cycler (BioRad, Netherlands). Values were normalized to β-actin. Primers are listed in table S1.

### Data processing and statistical analysis

The relative quantitative result (peak areas) of all the detected peaks was firstly normalized by IS (Myristic-1,2-^13^C2 acid). Partial least squares discriminant analysis (PLS-DA) were performed in the Metaboanalyst web portal (http://www.metaboanalyst.ca). Samples from the same groups were classified into one for PLS-DA modeling. Hierarchical clustering of signature metabolites altered in GW4064-treated subjects compared to control subjects was performed in MetaboAnalyst 2.0. Other experimental data are expressed as mean ± standard deiviation (SD). Differences between two groups were tested by the Student’s t test. Differences among multiple groups were tested using one-way analysis of variance followed by Dunnett’s *post-hoc* comparisons. Differences were considered significant if *P* < 0.05.

## Additional Information

**How to cite this article**: Xie, Y. *et al.* Farnesoid X receptor activation promotes cell proliferation via PDK4-controlled metabolic reprogramming. *Sci. Rep.* **6**, 18751; doi: 10.1038/srep18751 (2016).

## Supplementary Material

Supplementary Information
