# Supplementary material for: Farnesoid X receptor activation promotes cell proliferation via PDK4-controlled metabolic reprogramming
Source: Sci Rep. 2016 Jan 5;6:18751. doi: 10.1038/srep18751 (PMC4700422; doi:10.1038/srep18751)
Supplement: Supplementary Information [file srep18751-s1.pdf]

Farnesoid X receptor activation promotes cell proliferation via PDK4-controlled metabolic reprogramming

Yang Xie[1,2 #], Hong Wang[1#], Xuefang Cheng[1], Yuzheng Wu[1], Lijuan Cao[1], Mengqiu Wu[1], Wen Xie[2], Guangji Wang[1]*, Haiping Hao[1]*

[1]State Key Laboratory of Natural Medicines, Key Laboratory of Drug Metabolism and Pharmacokinetics, China Pharmaceutical University, Nanjing 210009, China.

[2]Center for Pharmacogenetics and Department of Pharmaceutical Sciences, University of Pittsburgh School of Pharmacy, Pittsburgh, Pennsylvania 15261, USA.

*Corresponding author: Telephone: +86-25-83271179, Fax: +86-25-83271060, Email: hhp_770505@hotmail.com (H. Hao) or guangjiwang@hotmail.com (G. Wang)

[#] These authors contribute equally to this work.

Supplementary Materials

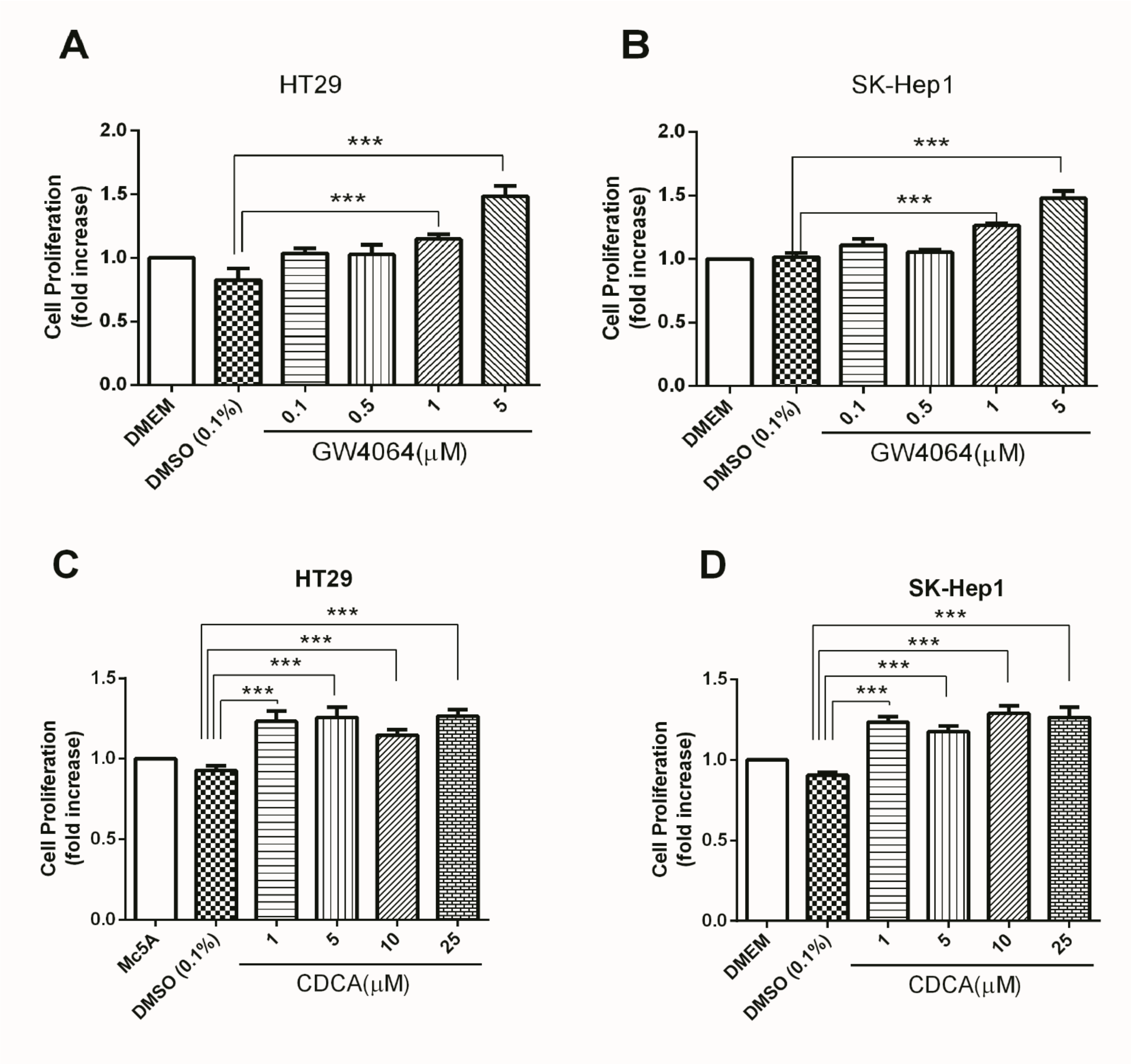

Fig. S1 FXR agonists trigger proliferation of cell lines. (A) HT29 cells were treated with different concentrations of GW4064. (B) SK-Hep1 cells were treated with different concentrations of GW4064. (C) HT29 cells were treated with different concentrations of CDCA. (D) SK-Hep1 cells were treated with different concentrations of CDCA. Values are presented with mean ± SD (n=6; *, $P < 0.05$;**,$P < 0.01$;***,$P < 0.001$).

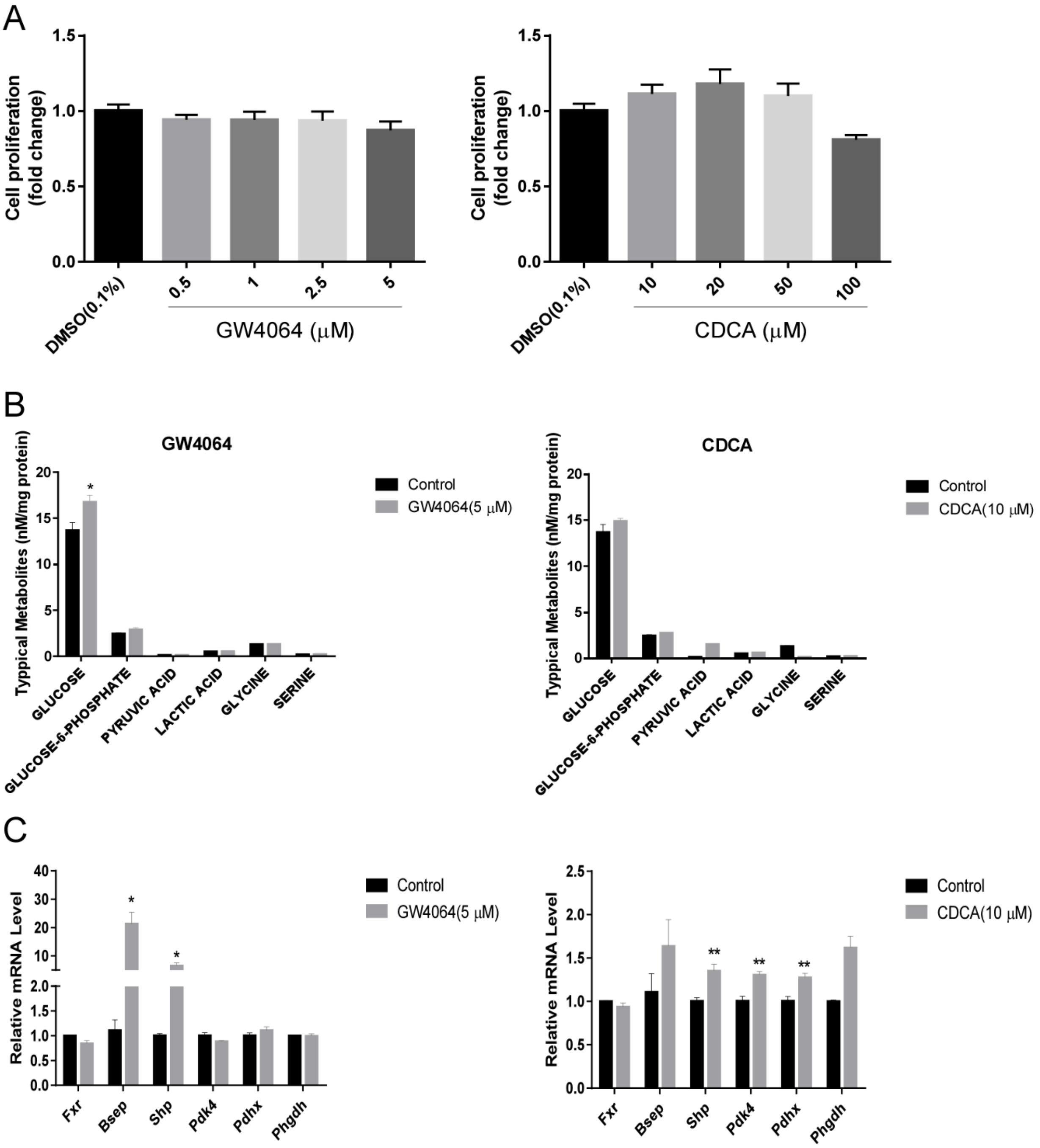

Fig. S2 FXR agonists failed in triggering proliferation of primary hepatocytes. (A) MTT assay on primary hepatocytes treated with GW4064 (Left) of CDCA (Right). (B) Targeted metabolomic analysis from primary hepatocytes treated with GW4064 (Left) of CDCA (Right). (C) RT-PCR in testing gene expression on primary hepatocytes after treatment with GW4064 (Left) of CDCA (Right). (n=6; *, $P < 0.05$;**,$P < 0.01$).

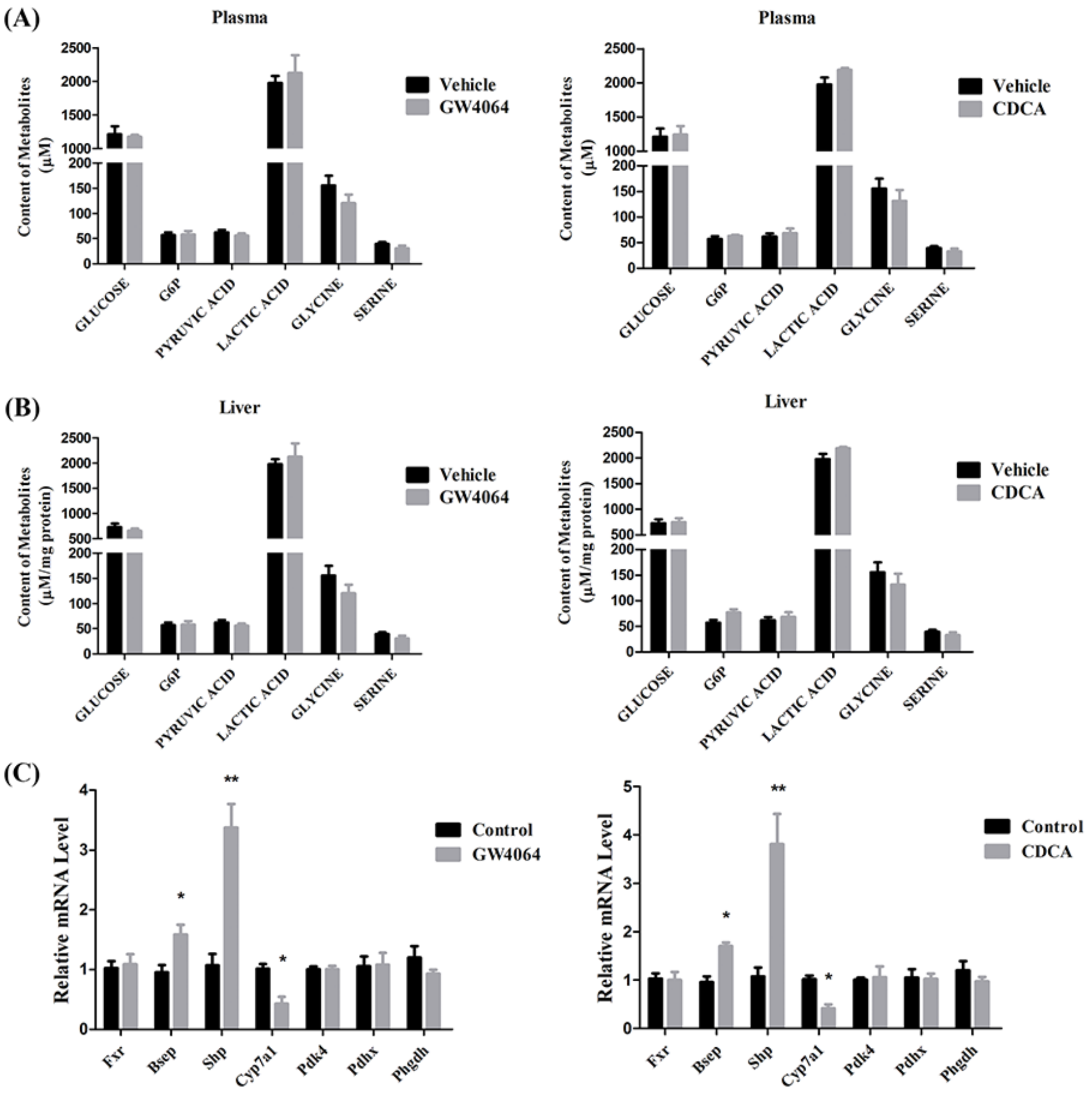

Fig. S3 FXR activation has little effect on glycolytic metabolism in the liver of healthy mice. Healthy mice were intraperitoneally administrated with GW4064 (30 mg/kg/day) or CDCA (100 mg/kg/day) for concentrative 3 days. Plasma and liver samples were collected. (A) Concentrations of major intermediates in glycolysis pathway in plasma. (B) Concentration of major intermediates in glycolysis pathway in liver. (C) Gene expression of FXR target genes, including Bsep, Shp, Cyp7a1, Pdk4, Pdhx and Phgdh in liver. Values are presented with mean ± SD (n=6; *, $P < 0.05$;**, $P < 0.01$).

Table. S1

Table.S1-1 Percent change(%) [b] of major metabolites in the HepG2 cell line (Treatment with GW4064)

Superpathway	Subpathway	Identified Metabolites	KEGG ID	Trend[a]	Cell lines		Trend[a]	Medium	
					HepG2	*p* value[c]		HepG2	*p* value[c]
**Carbohydrate metabolism**	Ascorbate and aldarate metabolism	D-Glucurono-6,3-lactone	C02670	↑	7.22				
	Ascorbate and aldarate metabolism	Threonic acid	C01620	↑	58.76				
	Citrate cycle (TCA cycle)	Citric acid	C00158		7.86		↓	-15.26	
	Citrate cycle (TCA cycle)	Fumaric acid	C00122	↑	30.05	*			
	Citrate cycle (TCA cycle)	Malic acid	C00149		31.56	*		15.68	
	Fructose and mannose metabolism	D-Fructose	C00095	↓	-7.51			-60.89	*
	Galactose metabolism	D-Galactose	C00124	↑	32.31	**		5.85	
	Galactose metabolism	Sucrose	C00089		2.44			17.78	
	Glycolysis / Gluconeogenesis	D-Glucose	C00031	↑	35.83	**	↓	-0.16	
	Glycolysis / Gluconeogenesis	Glucose 6-phosphate	C00668	↑	3.13			46.98	
	Glycolysis / Gluconeogenesis	L-Lactic acid	C00186	↑	9.05	**		4.00	
	Glycolysis / Gluconeogenesis	Pyruvic acid	C00022		-2.80			-2.28	
	Inositol phosphate metabolism	Myo-inositol 1-phosphate	C01177		-14.67	**		7.69	
	Pyruvate metabolism	Propylene glycol	C00583		-16.45	*		3.12	
**Nucleotide metabolism**	Purine metabolism	Adenine	C00147	↓	-14.05	*			
	Purine metabolism	Urea	C00086	↑	2.74		↓	-7.54	
**Amino acid metabolism**	Alanine, aspartate and glutamate metabolism	D-Aspartic acid	C00402		18.47	*		41.31	
	Alanine, aspartate and glutamate metabolism	L-Asparagine	C00152	↑	35.30	***			
	Alanine, aspartate and glutamate metabolism	L-Glutamic acid	C00025		15.89	*		1.04	
	Alanine, aspartate and glutamate metabolism	L-Glutamine	C00064	↑	50.43	***	↓	-1.16	
	Arginine and proline metabolism	L-Proline	C00148	↑	4.78			3.52	
	Arginine and proline metabolism	N-Acetylglutamic acid	C00624		15.89	*	↓	-4.80	
	Arginine and proline metabolism	Spermidine	C00315	↓	-62.37	**			

	Cyanoamino acid metabolism	Alanine	C01401	↑	18.47			1.23
	Cyanoamino acid metabolism	Cysteine	C00736	↑	42.41	***	↓	-5.12
	Glutathione metabolism	Putrescine	C00134	↑	304.71	***	↓	-14.08
	Glutathione metabolism	Pyroglutamic acid	C01879	↑	28.36	***	↓	-7.37
	Glycine, serine and threonine metabolism	Glycine	C00037	↑	10.77	*		41.81
	Glycine, serine and threonine metabolism	L-Cystathionine	C02291	↓	-6.17			
	Glycine, serine and threonine metabolism	L-Serine	C00065		-2.06		↓	-10.16
	Glycine, serine and threonine metabolism	L-Threonine	C00188	↑	58.76	**		3.08
	Glycine, serine and threonine metabolism	L-Tryptophan	C00078		18.71	**		2.45
	Lysine biosynthesis	L-Lysine	C00047		12.38	*		3.43
	Phenylalanine metabolism	L-Phenylalanine	C00079	↑	19.98	**	↓	-2.11
	Valine, leucine and isoleucine degradation	L-Isoleucine	C00407	↑	20.54	**		2.92
	Valine, leucine and isoleucine degradation	L-Valine	C00183	↑	18.47			0.54
**Lipid Metabolism**	Fatty acid biosynthesis	Oleic acid	C00712	↑	3.62		↓	-75.97
	Fatty acid biosynthesis	Stearic acid	C01530	↑	3.74		↓	-55.64
	Glycerolipid metabolism	Glycerol 3-phosphate	C00093		-6.96		↓	-61.75
	Steroid biosynthesis	Cholesterol	C00187	↑	14.01	*		-20.44

[a]Change trend of relative amounts of the GW4064 group compared to the Control group. (↑): up-regulated. (↓): down-regulated.

[b]The fold change of relative amounts of the GW4064 group compared to the Control group.

[c]Statistical p value calculated using the independent‘t’test (significance at $p < 0.05$)

Table.S1-2 Percent change(%) [b] of major metabolites in the HepG2 cell line (Treatment with CDCA)

	Subpathway	Identified Metabolites	KEGG ID	Trend[a]	Cell lines		Trend[a]	Medium	
					HepG2	*p* value[c]		HepG2	*p* value[c]
**Carbohydrate metabolism**	Carbon fixation pathways in prokaryotes	4-Hydroxybutyric acid	C00989		8.41				
	Citrate cycle (TCA cycle)	Citric acid	C00158		6.93			-3.02	
	Fructose and mannosemetabolism	D-Mannose	C00159		19.04			-1.48	
	Fructose and mannosemetabolism	D-Fructose	C00095	↑	19.04		↓	-12.19	
	Glycolysis / Gluconeogenesis	D-Lactic acid	C00256	↑	11.71	**		-2.98	*
	Glycolysis / Gluconeogenesis	D-Glucose	C00031	↑	46.59	**	↓	-1.78	*
	Glycolysis / Gluconeogenesis	Glucose 6-phosphate	C00668	↑	13.42	**		3.09	
	Glycolysis / Gluconeogenesis	Pyruvic acid	C00022		12.95	***		3.76	
	Inositol phosphate metabolism	Myoinositol	C00137		428.83	*	↓	-0.56	
	Pentose phosphate pathway	D-Ribose	C00121		-4.94			0.39	
	Pyruvate metabolism	Malic acid	C00149		-4.65			-37.05	*
**Nucleotide metabolism**	Purine metabolism	Hypoxanthine	C00262		31.25	*			
	Purine metabolism	Urea	C00086	↑	45.22	**		75.77	
	UMP biosynthesis	Uridine 5'-monophosphate	C00105		6.89				
**Amino acid metabolism**	Alanine, aspartate and glutamate metabolism	Alanine	C01401		25.20	**		8.17	**
	Alanine, aspartate and glutamate metabolism	L-Asparagine	C00152		25.65				
	Alanine, aspartate and glutamate metabolism	L-Aspartic acid	C00049		34.99	**		24.57	**
	Arginine and proline metabolism	Creatinine	C00791	↑	0.48			19.94	
	Arginine andprolinemetabolism	L-Proline	C00148		-6.88			1.18	
	Cysteine and methionine metabolism	Cysteine	C00736		-1.79			19.94	**
	Glycine, serine and threonine metabolism	Glycine	C00037		17.78			10.84	**
	Glycine, serine andthreoninemetabolism	L-Threonine	C00188		24.65	**		4.08	*
	Glycine, serine and threonine metabolism	L-Serine	C00065		22.81	**		19.66	*
	Lysine biosynthesis	L-Lysine	C00047		-3.93			2.51	
	Phenylalaninemetabolism	L-Phenylalanine	C00079		34.14	**		13.42	***
	Tyrosine metabolism	L-Tyrosine	C00082	↑	41.70			46.04	
	Tyrosine metabolism	N-Acetylglutamic acid	C00624		-24.91	*		-13.66	
	Valine, leucine and isoleucine degradation	N-Acetyl-L-aspartic acid	C01042		-71.12				
	Valine, leucine and isoleucine degradation	L-Valine	C00183		24.95	*		7.88	
	Valine, leucine andisoleucinedegradation	L-Isoleucine	C00407	↑	36.93	**		5.36	
**Lipid metabolism**	Biosynthesis of unsaturated fatty acids	Arachidonic acid	C00219	↑	8.05			7.79	
	Biosynthesis of unsaturated fatty acids	Oleic acid	C00712	↑	40.30	**		-49.61	
	Biosynthesis of unsaturated fatty acids	Palmitic acid	C00249		30.84	**		18.89	

	Biosynthesis of unsaturated fatty acids	Palmitoleic acid	C08362		16.97				
	Biosynthesis of unsaturated fatty acids	Stearic acid	C01530	↑	35.56	**		19.66	
	Glycerolipid metabolism	Propane-1,3-diol	C02457		1.75			-2.92	
	Glycerophospholipid metabolism	Glycerol 3-phosphate	C00093		-11.82			-13.63	
	Primary bile acid biosynthesis	Cholesterol	C00187		26.72	**			
**Metabolism of other amino acids**	D-Glutamineand D-glutamate metabolism	L-Glutamine	C00064	↑	26.74	*	↓	-12.19	
	D-Glutamine and D-glutamatemetabolism	L-Glutamic acid	C00025		11.54			31.57	**
	Glutathione metabolism	Cadaverine	C01672		40.43			19.94	
**Metabolism of cofactors and vitamins**	Nicotinate and nicotinamidemetabolism	Niacinamide	C00153	↑	-50.86			-49.61	
	Vitamin digestion and absorption	Pantothenic acid	C00864	↑	29.89	*		8.58	

[a]Change trend of relative amounts of the CDCA group compared to the Control group. (↑): up-regulated. (↓): down-regulated.

[b]The fold change of relative amounts of the CDCA group compared to the Control group.

[c]Statistical p value calculated using the independent‘t’test (significance at $p < 0.05$)

Table S2

Primer sequences for qRT-PCR(human)

Gene	Sequence	Locus
Glut1	TATGTGGAGCAACTGTGT	NM_006516.2
	GAAGTAGGTGAAGATGAAGAAC	
PFKL	GATGATGTTGGAGACGCTCA	NM_002626.4
	GGTGCCAAAGTCTTCCTCAT	
G3PDH	TATGACAACAGCCTCAAGAT	NM_001256799.1
	GAGTCCTTCCACGATACC	
PDK1	GGAGGTCTCAACACGAGGTC	NM_002610.3
	CGCTGGGTAATGAGGATTTG	
PDK4	CACGATGTGAATTGGTTGGT	NM_002612.3
	TGCCTTTGAGTGTTCAAGGA	
PKLR	GAGAAGTTGAGTCGCGCAAT	NM_181871.3
	CAGTACCAGCATCATTGCCA	
PKM	CTTCACTATGGGGCTTCGAC	NM_001206796.1
	TCAGAGAGAGGAGAACGGCT	
PDHX	AGGCTTACTTACTCCAATCA	NM_001166158.1
	GGTATTCTTCAGGCAACAAT	
HKI	CATTCGTAAGGTCCATTC	NM_000188.2
	CTCCATGTGAACATTCTG	
HKII	ACAATGGATGCCTAGATG	NM_000189.4
	AGGTACATTCCACTGATC	
FGFR4	ACAGCTCTCAGGGACCCAAG	NM_213647.1
	GCTGGAGCTGGGAGTGAG	
SHP	ACTTCACACAGCACCCAGTG	NM_021969.2
	AGGGACCATCCTCTTCAACC	
BSEP	CATTTCGCTCTCGATGTTCA	NM_003742.2
	TTCCAGGAAAAGCATGTGTG	
FXR	CACAGCGTTTTTGGTAATGC	NM_001206993.1
	TTGTTTGTGGAGACAGAGCCT	

PHGDH	TTCTCAGCTGCGTTGATGAC	NM_006623.3
	GCAAAGAGGAGCTGATAGCG	
FASN	TGAGGTTGTCCCAGAACTCC	NM_004104.4
	CTCCAGCCTCGCTCTCC	
ACTB	GTTGTCGACGACGAGCG	NM_001101.3
	GCACAGAGCCTCGCCTT	

Table S3

Primer sequences for qRT-PCR(mouse)

**Gene**	**Sequence**	**Locus**
Pdk4	TGACAGGGCTTTCTGGTCTT	NM_013743.2
	AGTGAACACTCCTTCGGTGC	
Pdhx	GTTCCCTTGCTCCATCGTAG	NM_175094.5
	GAGCAAGTTGGAGGTGGTTT	
Fgfr4	CAGGTCTGCCAAATCCTTGT	NM_008011.2
	CAGAGGCCTTTGGTATGGAT	
Shp	AGACTTCACACAGTGCCCAG	NM_011850.2
	AGCTGGGTCCCAAGGAGTAT	
Bsep	AAGGACAGCCACACCAACTC	NM_021022.3
	CCAGAACATGACAAACGGAA	
Fxr	GAAACTGAACATCGGGGTTAT	NM_001163700.1
	CGGCGGAGATTTTCAATAAG	
Phgdh	ACCTTCATCCACAATGCCTC	NM_016966.3
	GGAGATCTGGCCTCTCTGTG	
Fasn	ATGTCCACACCACCAATGAG	NM_007988.3
	CTCGCTTGTCGTCTGCCT	
Actb	ATGGAGGGGAATACAGCCC	NM_007393.3
	TTCTTTGCAGCTCCTTCGTT	

Table S4

Sequences of small interfering RNA

siRNA	Sequence
NR1H4	UCAGAGAUACCACUAUUUCGAAUUC
	GAAUUCGAAAUAGUGGUAUCUCUGA
PDK4	AUAAAGAGUAGAGAUUCAGAUCUCC
	GGAGAUCUGAAUCUCUACUCUUUAU

Methods

Primary hepatocyte isolation, culture and treatment

Primary mouse hepatocytes were isolated following a two-step in situ collagenase perfusion method as described previously. Hepatocytes were seeded at subconfluence (0.5 × 106/ ml) in Williams E Medium (Gibco, Life Technologies, Carlsbad, CA) supplemented with 10 % (v/v) fetal bovine serum (Hyclone, Logan, Utah), 0.1 μM dexamethasone, 10 μg/ml insulin, 2 mM L-glutamine, and 100 μg/ml penicillin and streptomycin in collagen I-coated cell plate. Four hour after incubation, the cells were washed and incubated in serum-free Williams E Medium. Hepatocytes were cultured at 37 ℃, 5 % CO2, and 95% relative humidity. After 24 h in serum-free cultivation medium, hepatocytes were washed with PBS and were stimulated with GW4064 and CDCA for another 24 h.

For cell proliferation assay, primary mouse hepatocytes were seeded in 96-well plates and treated with GW4064 at concentrations of 0.5, 1, 2.5, and 5 μM or CDCA at concentrations of 10, 20, 50, and 100 μM for 24 h. Cell proliferation was tested through MTT method. For gene expression and metabonomics analysis, hepatocytes were seeded in 6-well plates and treated with GW4064 and CDCA at the concentration of 5 or 10 μM for 24 h.

Animals and Treatments

Specific pathogen free (SPF) male C57BL/6 mice (8 wk old, 20 g) were obtained from Comparative Medicine Centre of Yangzhou University, China. The animal studies were approved by the Animal Ethics Committee of China Pharmaceutical University and have been carried out in accordance with the Declaration of Helsinki. Animals were housed in an air-conditioned room (25 ℃) under a 12 h light/dark cycle for 1 week before experiments and allowed water and standard chow *ad libitum*. Mice were treated with GW4064 (30 mg/kg/day, *i.p*) or CDCA (100 mg/kg/day, *i.p*) once a day for concentrative 3 days. 24 h after the last dose, liver and plasma were collected and kept at -80 ℃ for further analysis. Concentrations of major intermediates in glycolysis pathway in plasma and liver were detected by GC-MS as described above. mRNA levels of main FXR target genes in liver were detected by RT-PCR method.
